# Loss of the crumbs cell polarity complex disrupts epigenetic transcriptional control and cell cycle progression in the developing retina

**DOI:** 10.1002/path.6056

**Published:** 2023-02-09

**Authors:** Nicholas Owen, Maria Toms, Yuan Tian, Lyes Toualbi, Rose Richardson, Rodrigo Young, Dhani Tracey‐White, Pawan Dhami, Stephan Beck, Mariya Moosajee

**Affiliations:** ^1^ UCL Institute of Ophthalmology University College London London UK; ^2^ The Francis Crick Institute London UK; ^3^ Medical Genomics, UCL Cancer Institute University College London London UK; ^4^ Department of Ophthalmology Great Ormond Street Hospital for Children NHS Foundation Trust London UK; ^5^ Department of Genetics Moorfields Eye Hospital NHS Foundation Trust London UK

**Keywords:** polarity complex, retina, transcriptome, RNA‐seq, epigenome, DNA methylation, zebrafish, iPSC

## Abstract

The crumbs cell polarity complex plays a crucial role in apical–basal epithelial polarity, cellular adhesion, and morphogenesis. Homozygous variants in human *CRB1* result in autosomal recessive Leber congenital amaurosis (LCA) and retinitis pigmentosa (RP), with no established genotype–phenotype correlation. The associated protein complexes have key functions in developmental pathways; however, the underlying disease mechanism remains unclear. Using the *oko meduzy* ^*m289/m289*^ (*crb2a* ^*−/−*^) zebrafish, we performed integrative transcriptomic (RNA‐seq data) and methylomic [reduced representation bisulphite sequencing (RRBS)] analysis of whole retina to identify dysregulated genes and pathways. Delayed retinal cell specification was identified in both the *crb2a* ^*−/−*^ zebrafish and *CRB1* patient‐derived retinal organoids, highlighting the dysfunction of cell cycle modulation and epigenetic transcriptional control. Differential DNA methylation analysis revealed novel hypermethylated pathways involving biological adhesion, Hippo, and transforming growth factor β (TGFβ) signalling. By integrating gene expression with DNA methylation using functional epigenetic modules (FEM), we identified six key modules involving cell cycle control and disturbance of TGFβ, bone morphogenetic protein (BMP), Hippo, and SMAD protein signal transduction pathways, revealing significant interactome hotspots relevant to *crb2a* function and confirming the epigenetic control of gene regulation in early retinal development, which points to a novel mechanism underlying *CRB1*‐retinopathies. © 2023 The Authors. *The Journal of Pathology* published by John Wiley & Sons Ltd on behalf of The Pathological Society of Great Britain and Ireland.

## Introduction

Genetic variants in Crumbs cell polarity complex component 1 gene [*CRB1*, Online Mendelian Inheritance in Man (OMIM) No. 604210] cause a heterogenous spectrum of retinopathies; accounting for 7–17% of all Leber congenital amaurosis (OMIM No. 613935, LCA8), 3–9% of autosomal recessive retinitis pigmentosa (RP) (OMIM No. 600105, RP12), autosomal dominant pigmented paravenous chorioretinal atrophy (OMIM No. 172870), and, rarely, a cone‐rod or macular dystrophy [1, 2, 3, 4, 5, 6, 7, 8, 9]. LCA8 is an early‐onset severe retinal dystrophy presenting in infancy with nystagmus, poor vision, and a non‐detectable electroretinogram, with no existing treatment. The retina is characteristically thickened with loss of retinal lamination [10]. RP presents with primary rod photoreceptor degeneration, followed by secondary cone loss, and symptoms include night blindness followed by concentric peripheral visual field loss with eventual central vision loss. More than 300 causative variants have been reported in *CRB1*‐associated retinopathy with no clear genotype–phenotype correlation [3, 11]. Recent advances in 3D retinal organoid (RO) cultures derived from three *CRB1* patient‐induced pluripotent stem cells (iPSC) have shown frequent outer limiting membrane (OLM) integrity defects at day 160 [12]. Day 35 RO reported *CRB1* alternative splicing, though no overt phenotype was observed [13]. The underlying mechanism determining how genetic variants in *CRB1* produce RP or LCA phenotypes remains unknown, although *CRB2* may function as a modifier [14].

Since the discovery of the crumbs (*crb*) gene in *Drosophila*, three *CRB* orthologues have been found in mammals [15, 16]. Human and mouse *CRB1* are expressed specifically in the brain and eye and are required for normal development and function of the retina, and particularly photoreceptor survival [1, 17, 18]. The gene encodes a type I transmembrane protein consisting of 19 epidermal growth factor (EGF) domains, three laminin‐globular (LamG)‐like extracellular domains, and a short FERM/PDZ binding motif containing an intracellular cytoplasmic tail, which localises in mammals to the subapical region of Müller glia and photoreceptor cells. Recently a novel *CRB1* transcript (*CRB1‐B*) was identified and shown to be the dominant isoform in retina [19]. *CRB2* is predominantly expressed in the foetal eye, retinal pigment epithelium (RPE), and choroid, as well as in the brain and kidney [20]. Pathogenic variants in *CRB2* have been identified in non‐syndromic autosomal recessive RP [21], with *CRB2* playing a crucial role in photoreceptor survival [22], as well as syndromic kidney and brain diseases [23]. *CRB3* is expressed in all epithelial tissues, but it lacks the extracellular EGF repeats and LamG regions of other CRB proteins [24].

The CRB extracellular region plays critical roles in intercellular adhesion and NOTCH signalling [25, 26]. The short intracellular domains enable CRB to interact with several protein complexes regulating epithelial apicobasal polarity, Hippo/YAP signalling, and actomyosin organisation [27, 28, 29]. Apicobasal polarity is established and maintained by several interconnected complexes; Crumbs‐PALS‐PATJ and PARD3‐PARD6‐aPKC localise to the apical membrane domain and promote its formation [30, 31, 32]. A recent study into the apical border of epithelial cells identified a novel polarity domain apical of tight junctions (TJ), the vertebrate marginal zone (VMZ) defined by the Crumbs complex [33]. The Crumbs‐PALS‐PATJ complex is critical to the formation of epithelial tight or adherens junctions (AJs) [34, 35, 36, 37], and a loss of CRB results in disruption within the retinal neuroepithelium showing cellular adhesion defects at the level of the OLM [38]. These polarity complexes are not separate entities but enriched protein–protein interaction (PPI) networks that co‐regulate each other's localisation and regulation.

To further explore the function of crumbs in the developing retina, we used the *oko meduzy* ^*m289*^ zebrafish model with a nonsense variant c.764A>T, p.(Arg1466*) in exon 8 of *crb2a* [39, 40]. Zebrafish have five *crb*‐related genes (*crb1*, *crb2a*, *crb2b*, *crb3a*, *crb3b*) resulting from a partial genomic duplication [40]. Mutant studies have indicated that *crb2a* is most functionally orthologous with human *CRB1* in the retina [40, 41]; the *crb2a* null develops a severe retinal phenotype in the embryonic stages closely resembling the human condition *CRB1‐*LCA, with loss of retinal lamination and retinal thickening. In contrast, the *crb1* null showed no abnormality in development, surviving into adulthood, with no disruption to retinal patterning and morphology [42]. *crb1* is localised to the cell membranes surrounding the axonemes in cone outer segments. Retinal progenitor cells (RPCs) lose polarity and AJ function in *crb2a* ^*−/−*^, resulting in cell detachment, which has been suggested to cause severe retinal laminae disorganisation [43]. We investigated the molecular changes in the retina at 56 h post‐fertilisation (hpf) compared to age‐matched wild‐type (WT) AB controls. We highlight a pathological level of cell proliferation within the retina, where cellular differentiation is inhibited or paused, which was also substantiated in *CRB1*‐LCA ROs. In addition, significant perturbations to epigenetic pathways were detected, so we undertook an analysis of DNA methylation (DNAm) via high‐throughput reduced representation bisulphite sequencing (RRBS) [44] and found a global correlation between the RNA‐seq expression and methylation status, acquiring new insights into the molecular pathophysiology of *CRB1*‐related disease.

## Materials and methods

### Ethical approval

This study was approved by Moorfields Eye Hospital and the National Research Ethics Committee and was conducted in adherence to the tenets of the Declaration of Helsinki. Informed consent was obtained from all subjects or patients’ legal guardians through the Genetic Study of Inherited Eye Disease (REC reference 12/LO/0141).

### Animal husbandry

WT strain AB and mutant *oko meduzy* (*crb2a* ^*m289*^) zebrafish were bred and maintained according to local UCL and UK Home Office regulations under the Animals Scientific Procedures Act (License No. PPL PC916FDE7). All approved standard protocols followed the guidelines of the ARVO Statement for the Use of Animals in Ophthalmic and Vision Research Ethics (https://www.arvo.org/About/policies/statement‐for‐the‐use‐of‐animals‐in‐ophthalmic‐and‐vision‐research/, last accessed January 2022).

### RNA isolation and RNA‐seq analysis

Dorsal retina (DR) was dissected from 56 hpf *crb2a* ^−/−^ and WT zebrafish (*n* = 6 per group). Staging of the animals and RNA isolation were carried out as previously described [45]. In brief, sequencing libraries were generated from high‐quality RNA using a SMART‐Seq v4 Ultra Low Input RNA Kit for Sequencing (Takara Bio, London, UK). Paired‐end reads were preprocessed for quality, aligned to the *Danio rerio* reference annotation (GRCz11, version 95), and gene count data were used for differential expression analysis (DESeq2 [46]). False discovery rate (FDR) was controlled by Benjamini–Hochberg (BH) multiple testing correction. All analysis was conducted at a detection level with an alpha of 0.05, adjusted *p* value of ≤0.05, and absolute log2 fold‐change (LFC) ≥1 for differentially expressed genes (DEGs). GO‐term enrichment was carried out using gProfiler [47], enriched pathways, with a FDR *p* value ≤0.05 selected.

### RT‐qPCR

Total RNA from 10 ROs from three independent rounds of differentiation was isolated using RNeasy Plus Mini Kit (Qiagen, Hilden, Germany; Catalogue No. 74104) following the manufacturer's instructions. For zebrafish samples, dissected retinas were pooled (*n* = 10) for RNA preparation using an RNeasy FFPE kit (Qiagen; Catalogue No. 73504). cDNA was generated using a High‐Capacity RNA‐to‐cDNA™ Kit (Thermo Fisher Scientific, Waltham, MA, USA; Catalogue No. 4387406), quantitative PCR (Applied Biosystems 7500 Fast Real‐Time PCR System, Thermo Fisher Scientific; Catalogue No. 4406984) using SYBR™ Green PCR Master Mix (Thermo Fisher Scientific; Catalogue No. 4364344) and 0.2 μm forward/reverse primers (supplementary material, Table S1) in triplicate. Cycles were 95 °C for 30 s, 40 × 95 °C for 5 s, and 60 °C for 30 s. All data were normalized to the internal reference transcript (*GAPDH* for human and *rpl13a* for zebrafish) with fold‐changes calculated using 2^−ΔΔCT^.

### Quantification of S‐ and M‐phase retinal progenitor cells

Analysis of proliferating cells was undertaken using either the Click‐iT EdU cell proliferation kit (Invitrogen, Waltham, MA, USA) for S‐phase or anti‐pH3 immunohistochemistry for M‐phase analysis. ROs were treated following the manufacturers' protocols (*n* = 9). Zebrafish embryos were placed in 500 μl of 500 μm EdU in 15% DMSO/E3 medium for 30 min on ice, fixed in 4% paraformaldehyde (PFA), and briefly permeabilised (*n* = 8). Embryos were incubated in Click‐iT EdU reaction mixture for 1 h in the absence of light. Embryos were washed in PBS + 0.1% Tween‐20 and processed for immunostaining.

### Reduced representation bisulphite sequencing and data analysis

Dissected retinal samples (*n* = 10 per condition, pooled) were incubated for 3 h at 55 °C in DNA extraction buffer [10 mm Tris (pH 8.2), 300 mm NaCl, 0.5% SDS, 200 μg/μl proteinase K, 10 mm EDTA]. Genomic DNA was precipitated in two volumes of 100% ethanol, and samples were incubated on ice for 30 min before centrifugation at 16,000 × *g* for 20 min. After removal of supernatant, 100 μl of 70% ethanol was added and samples centrifuged for a further 2 min. All liquid was removed, and pellets were air‐dried before resuspension in nuclease‐free H_2_O. RRBS libraries were generated using Ovation RRBS Methly‐Seq (Tecan, Männedorf, Switzerland), and data were generated following the Nugen RRBS preprocessing pipeline, followed by beta‐value calculation and differential status for all CpG sites covered (methylKit).

### iPSC reprogramming and RO differentiation

A skin biopsy was obtained from a 12‐year‐old female patient carrying a compound heterozygous variant c.2548G>A p.(Gly850Ser) and c.4006‐10A>G in the *CRB1* gene and a healthy control (WT). Human dermal fibroblasts were reprogrammed using integration‐free episomal vectors: pCXLE‐hOCT3/4‐shp53‐F (Addgene, Watertown, MA, USA; Catalogue No. 27077), pCXLE‐Hsk (Addgene; Catalogue No. 27078), pCXLE‐Hul (Addgene; Catalogue No. 27080), and pCXWB‐EBNA1 (Addgene; Catalogue No. 37624) as previously described [48]. hiPSC were differentiated towards a retinal lineage [49]; briefly, hiPSCs were grown to 80–90% confluency (day 0), mTesR™ Plus was replaced by Essential 6™ (Thermo Fisher Scientific; Catalogue No. A1516401). From day 2 to day 28, differentiating cells were subjected to a neural induction medium Essential 6™, 1% N2 supplement (Stemcell Technologies, Cambridge, UK; Catalogue No. 17502048) and 0.1% Pen/Strep. By 3–4 weeks, neural retina‐like structures emerged from the cell layer and were manually dissected at day 28 using a 21G needle and further cultured until day 35 in maturation media [DMEM/F12 (Thermo Fisher Scientific; Catalogue No. 21331020), 1% MEM NEAA (Thermo Fisher Scientific; Catalogue No. 10370021), 2% B27 supplement (Thermo Fisher Scientific; Catalogue No. 12587010), 10 ng/ml FGF2 (Thermo Fisher Scientific; Catalogue No. 100‐18B), and 0.1% Pen/Strep].

### Sections and immunostaining

Day 35 ROs were washed in PBS and fixed in 4% PFA for 15 min at 4 °C, incubated in 30% sucrose:PBS solution overnight at 4 °C, then embedded in 7.5% gelatin:10% sucrose:PBS. Blocks mounted in optimal cutting temperature compound (OCT) (VWR International, Arlington Heights, IL, USA) were then frozen in isopentane at −50 °C. Cryosections were cut at a thickness of 10 μm and then processed and permeabilised for immunostaining. Sections were incubated with primary antibodies overnight at 4 °C, washed (Tween 0.2%:PBS), and incubated with fluorochrome‐conjugated secondary antibodies for 1 h at room temperature (RT). Sections were mounted using ProLong™ Diamond Antifade with DAPI (Invitrogen; Catalogue No. P36962), and images were acquired on a Leica LSM 710 confocal microscope and processed using ImageJ (https://imagej.net/ij/, last accessed 1 May 2022). For histological evaluation of zebrafish retina, fresh enucleated eyes were fixed with 4% PFA/PBS overnight at 4 °C, followed by incubation in 30% sucrose/PBS for 6 h at RT before processing and embedding using the JB‐4™ embedding kit (Polysciences, Warrington, PA, USA) with 7‐μm‐thick sections. Sections were imaged on a Leica DMRB with Jenoptik D‐07739 Optical System (Leica Microsystems, Milton Keynes, UK).

Primary antibodies utilised: anti‐PAX6 (1:100, BioLegend, San Diego, CA, USA; Catalogue No. PRB‐278P, RRID AB_291612), anti‐VSX2 (1:200, Santa Cruz Biotechnology, Santa Cruz, CA, USA; Catalogue No. sc‐365,519, RRID AB_10842442), anti‐BRN3 (1:300, Abcam, Cambridge, UK; Catalogue No. ab56026, RRID AB_880587), anti‐zpr1 (1:1000, Zebrafish International Resource Center, Eugene, OR, USA; Catalogue No. zpr‐1, RRID AB_10013803), anti‐GS (1:100, Abcam; Catalogue No. ab73593, RRID AB_2247588), anti‐MPP5 (1:200, Sigma‐Aldrich, St. Louis, MO, USA; Catalogue No. SAB2101502, RRID AB_10605917), anti‐YAP1 (1:200, Thermo Fisher Scientific; Catalogue No. PA146189, RRID AB_2219137), and anti‐crb (1:200, gift of the Malicki group).

### Statistics

DEG analysis was performed using the DESeq2 R package. Data are presented as mean ± SEM (*n* indicates the number of tissue preparations, cells, or separate experiments, as indicated). The statistical significance of differences between the means was evaluated using a two‐tailed Student's *t*‐test. All statistical tests were two‐sided, and statistical significance was considered at *p* < 0.05. R (version 3.6.1, https://www.R-project.org, last accessed 1 March 2022) was used for all statistical analyses.

## Results

### Loss of *crb2a* results in loss of retinal cell specification and increased cell proliferation

**Figure 1:**
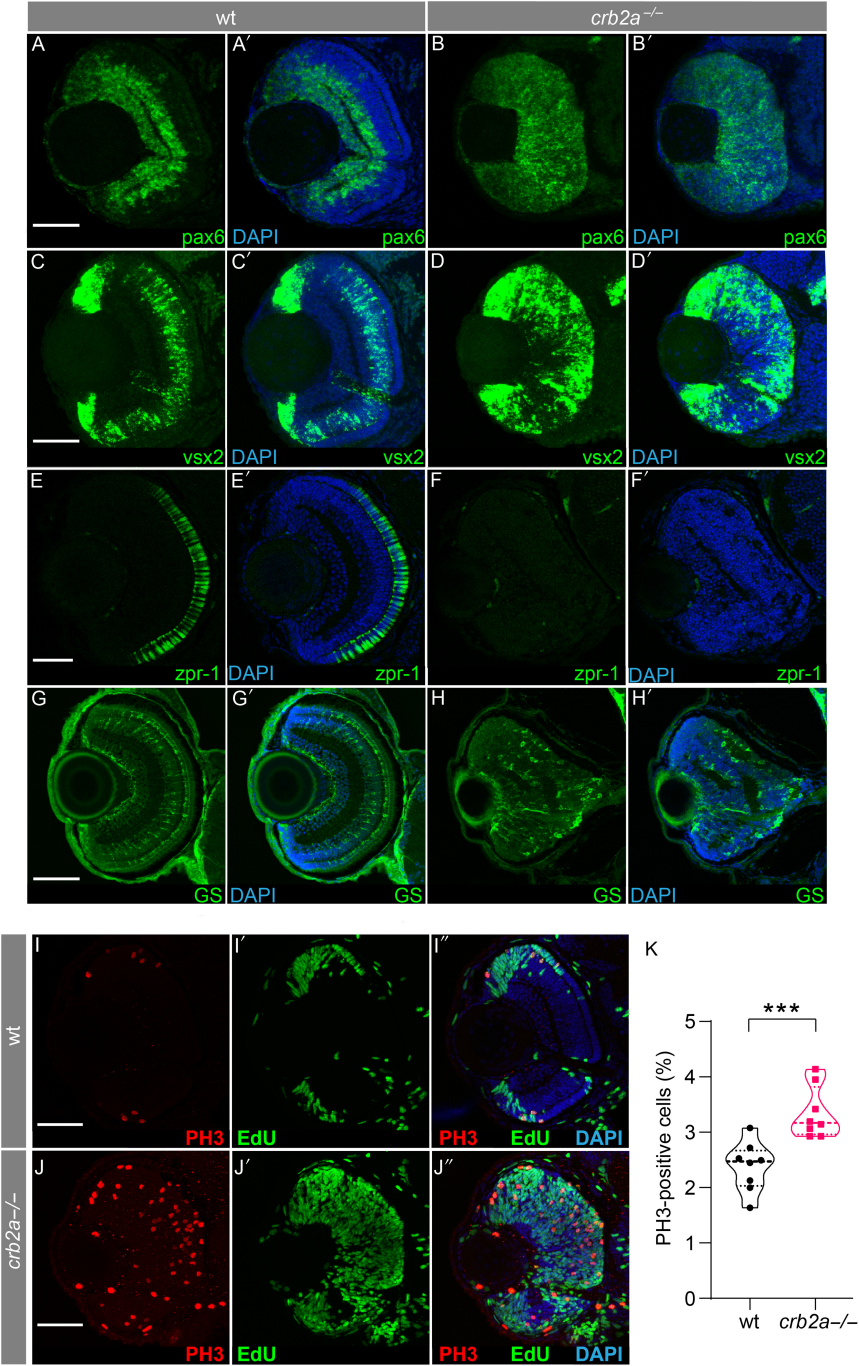
The effect of *crb2a* ^−/−^ on retinal neurogenesis and lamination. Characterisation of cell types present in zebrafish retina at 56 hpf (A–H, wild type zebrafish, A′‐H′ *crb2a* ^*m289*^ zebrafish). Early retinal progenitor cells were identified through staining with pax6 in WT (A and B) and *crb2a* ^−/−^ (A′ and B′) and anti‐vsx2 (C, D, C′ and D′) at 56 hpf. Anti‐ZPR1 antibody (zpr1) was used to identify the presence of cone cells (E and F) in WT; however these were absent from the mutant retina (E′ and F′) at 80 hpf. The presence of Müller cells was visualised using an anti‐glutamate synthetase antibody (GS) in WT (G and H) and mutant (G′ and H′) retina. M‐phase nuclei, visualised using an anti‐phospho‐Histone 3 antibody (PH3), were observed in both WT (I) and *crb2a* ^−/−^ (J) at 56‐hpf. All nuclei are stained with DAPI. S‐phase nuclei as visualised by 5‐ethynyl‐2’‐deoxyuridine (EdU) incorporation were observed in WT (I′) and *crb2a* ^−/−^ (J′) retina. Panels I″ and J″ are merged images of previous panels. (K) Quantification of PH3‐positive cells; *** *p* < 0.001. Scale bar, 50 μm.

To characterise alterations in neural retina development resulting from the loss of the crumbs‐associated complex, we examined the retinal organisation of homozygous null *crb2a* zebrafish (*crb2a* ^*m289/m289*^, termed *crb2a* ^*−/−*^). Histological analysis at 48–96 hpf showed an absence of retinal layer demarcation and complete cellular disorganisation, with patches of plexiform matter apparent from 56 hpf (supplementary material, Figure S1A) as compared to control retina with increased definition of retinal cell layers from 48 hpf onwards. Loss of *crb2a* was confirmed at 56 hpf compared to WT, which showed localisation in the OLM (supplementary material, Figure S1B). Increased expression of vsx2 and pax6, essential transcription factors for maintenance of proliferative states of RPC, was seen throughout the *crb2a* ^−/−^ retina at 56 hpf, indicating an expanded population of cells at early stages of retinal neurogenesis (Figure 1B,D). This was in contrast to WT retina, where pax6 was localised to the inner nuclear layer (INL) and ganglion cell layer (GCL) (Figure 1A), and vsx2 was highly expressed at the ciliary marginal zone (CMZ) and bipolar cells in the INL and outer plexiform layer (OPL) (Figure 1C). zpr‐1, a key marker for cone photoreceptors, was absent in the *crb2a* ^−/−^ at 80 hpf when neurogenesis was complete (Figure 1F) [50] compared to WT (Figure 1E). The presence of Müller glia was confirmed using glutamine synthetase expression at 4 dpf (Figure 1G,H), but the appearance was patchy and irregular in *crb2a* ^−/−^. No difference in apoptotic cell death was observed between the *crb2a* ^−/−^ and WT retina (data not shown).

To assess proliferative states, we used whole mount immunohistochemistry for the mitotic marker Phospho‐Histone 3 (PH3), indicating the onset of mitosis (M‐phase), and EdU incorporation as an S‐phase marker. In WT retina, M‐ and S‐phase nuclei were mostly concentrated at the CMZ (Figure 1I). However, an increased number of PH3‐positive cells were present throughout the *crb2a* ^*−/−*^ retina (Figure 1J), with striking EdU staining showing a significant increase in S‐phase nuclei (Figure 1J′). PH3‐positive cells were counted, showing a significant increase between *crb2a* ^−/−^ and WT controls (Figure 1K, *p* value 0.0008***). Considered together the results highlight the continued proliferative state, reduced cell‐cycle exit, and lack of neuronal differentiation, which correlates with mouse studies [51, 52].

**Figure 2:**
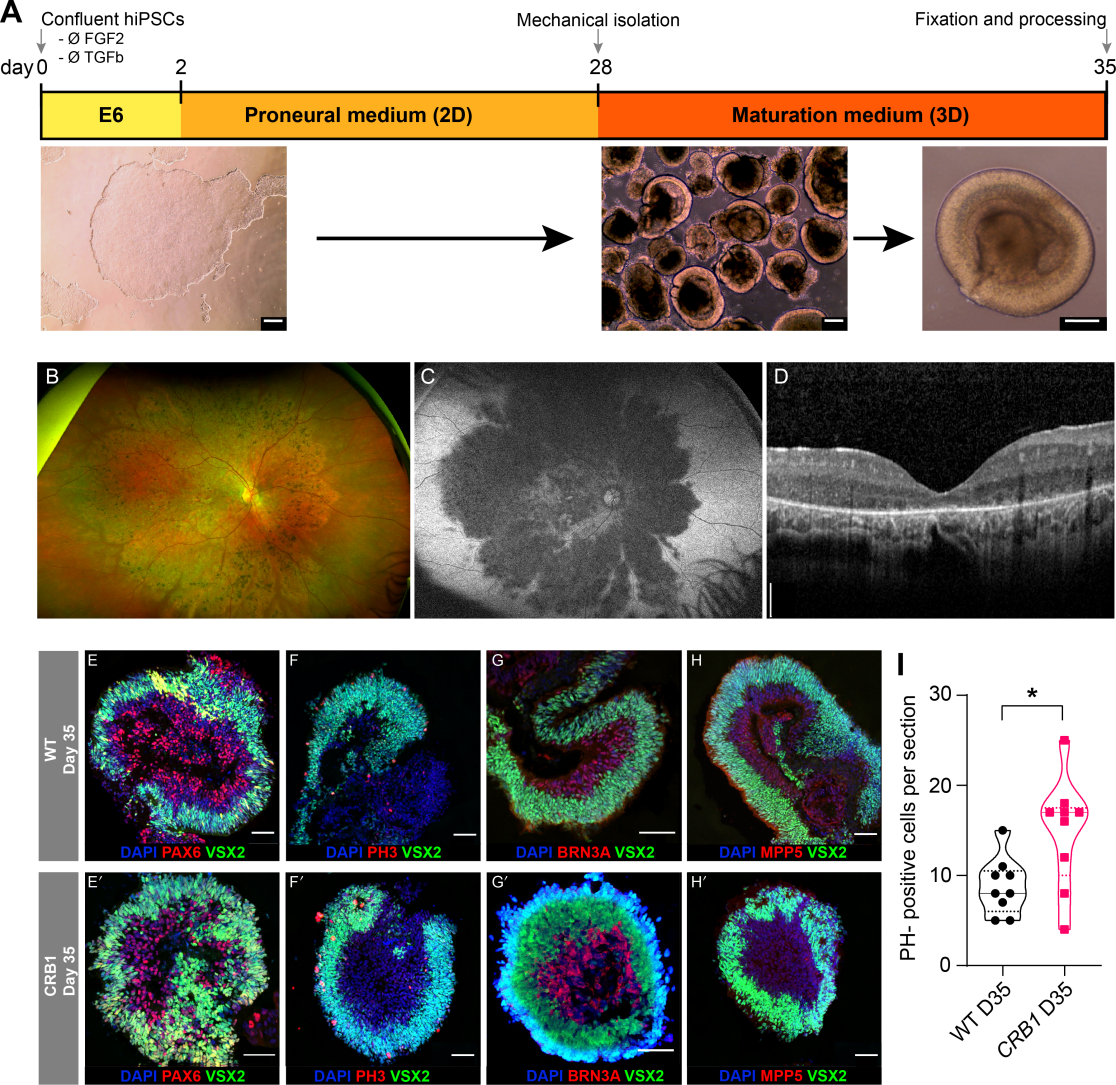
Retinal organoids of a *CRB1* patient. Clinical retinal imaging of a patient with *CRB1*‐LCA8 and generation of ROs from patient‐derived hiPSC. (A) Schematic of retinal differentiation 2D/3D protocol from hiPSC. (B) Fundus photograph, (C) fundus autofluorescence, and (D) retinal imaging using spectral domain‐optical coherence tomography of right eye of patient with *CRB1*‐LCA8. The nasal outer macula subfield was 414 μm thick with no signs of any oedema. Immunostaining images of WT and *CRB1* RO sections at day 35 for (E) PAX6/VSX2, (F) PH3/VSX2, (G) BRN3A/VSX2, and (H) MPP5/VSX2 expression. (I) Quantification of PH3‐positive cells per section analysed; * *p* < 0.02. Scale bars: A, 100 μm; D, 200 μm; E–H, 50 μm.

To validate our findings in human tissue, we generated human ROs from a 12‐year‐old female patient with molecularly confirmed *CRB1*‐LCA8 and a healthy control (WT, 28‐year‐old male) (Figure 2A) [49]. Retinal imaging of the patient showed right eye RPE atrophy and pigmentary changes (Figure 2B), as well as fundus autofluorescence indicative of RPE loss (hypoautofluorescence) but with preservation of the para‐arteriolar RPE (PPRPE) (Figure 2C). Increased retinal thickness and unlaminated architecture were also identified (Figure 2D), with the patient's mean macula volume (using the Early Treatment Diabetic Retinopathy Study [ETDRS] grid) from their Optimal Coherence Tomography being 10.04 mm^3^. The nasal outer macula subfield was 414 μm thick with no signs of any oedema. We performed immunostaining on day 35 ROs to compare RPC abundance, cell differentiation/proliferation, and MPP5 (PALS1) expression. Increased expression of VSX2 and PAX6 in *CRB1* RO suggested an enriched RPC population (Figure 2E), and increased PH3‐positive cells also indicated a significantly higher progenitor population with inhibited cell fate progression (Figure 2F,I, *p* value 0.017). Additionally, BRN3A‐positive retinal ganglion cells (RGCs) were reduced in abundance (Figure 2G), and there was complete absence of MPP5 (PALS1), a CRB1‐associated protein (Figure 2H), consistent with our findings in the zebrafish model.

### Transcriptional dysregulation in *crb2a* ^−/−^

**Figure 3:**
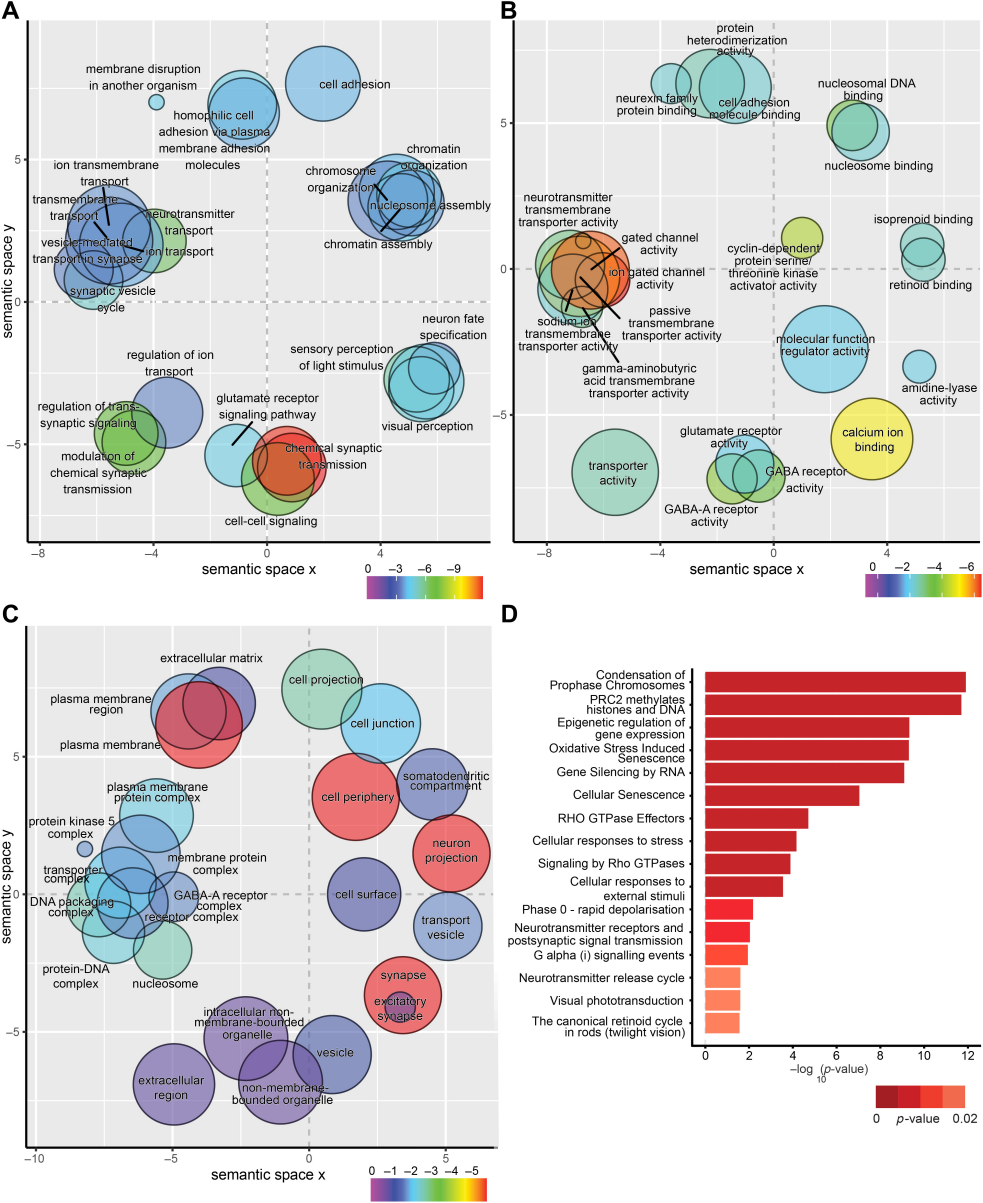
Gene ontology (GO) overrepresentation analysis of differentially expressed genes in *crb2a* ^*−/−*^ retina. Scatter plots of ontology enrichment analysis coloured by log_10_(*p*‐value) scores plotted in semantic space. The size of each point represents the number of genes overlapping the ontology, scaled. Multidimensional scaling (MDS) was used to reduce the dimensionality of the resulting GO terms’ semantic similarities, with the closeness in the plot reflecting the closeness in the GO directed acyclic graph structure. Thus, semantically similar terms should remain close together in the plot. (A) Biological process (GO:BP) ontology analysis, (B) molecular function (GO:MF) analysis, (C) cellular component (GO:CC), and (D) REACTOME pathway analysis.

To investigate how the loss of *crb2a* influenced the transcriptional state of the retina, we performed RNA‐seq on tissue isolated from *crb2a* ^*−/−*^ and WT zebrafish at 56 hpf. Principal component analyses (PCA) demonstrated distinct cluster separation, with PC1 representing approximately 60% of the component of sample differences (supplementary material, Figure S2A). We identified 1,242 DEG events upregulated in *crb2a* ^−/−^ and 1,568 DEGs downregulated compared to the control, shown by the volcano plot (supplementary material, Table S2 and Figure S2B). Gene ontology (GO) overrepresentation analysis identified top ontologies for biological process (GO:BP), including membrane disruption in other organisms and sensory perception of light stimulus, molecular function (GO:MF) highlighted ligand or voltage‐gated ion, cation, channel activities, and calcium ion binding, and cellular component (GO:CC) plasma membrane bounded cell projection and adherens junction (Figure 3A–C). Key biological processes involving Crumbs were identified providing validation of the approach, including cellular adhesion, ion transport, cellular communication, membrane transport, components of cellular projection, and plasma membrane. Reactome pathway analysis identified epigenetic regulation of gene expression, chromosome condensation, methylation, and visual transduction (Figure 3D).

### Apical–basal polarity complexes dysregulated

We identified prominent changes between key interactors of the Crumbs proteins *Pals1*/*MPP5*, *epb41L5*/*Moe*, *Mupp1*/*Patj*, *Par6*, *aPKC*, *patj*, *amot*, *lin7a*, and *notch1*, as well as components of the apical–basal polarity complexes: the Par complex (*cdc42*, *Par6*, *Par3*, *Tiam1*, *aPKC*) and Scribble complex (*Lgl*, *Scrib*, *Dlg*) (supplementary material, Figure S3). Crumbs has a direct inhibitory effect on notch, whose loss results in a significant increase in *notch1* (LFC 1.3, adjusted *p* value 1 × 10^−5^). Interestingly *epb41l5*/*moe*, known to reverse crumbs inhibition of notch, was not identified as differentially expressed, suggesting that the modulation of the crumbs complex function in *crb2a* ^−/−^ retina is not through *epb41l5* expression.

Unexpectedly, *mpp5a/b* genes were downregulated in *crb2a* ^−/−^ retina (LFC ‐1, −2.8 respectively); correspondingly, mpp5 (pals1) was undetectable in the retina (supplementary material, Figure S4A) and globally in the developing embryo (supplementary material, Figure S4B). Other components of the crumbs/mpp5 apicobasal protein complex, including *lin7a*, *prkci*, and *patj*, which also have a regulatory role, were reduced, but there was no evidence of any transcriptional feedback loops (supplementary material, Figure S3). The interconnected loss of mpp5 from functional knockout of *crb2a* has not been previously reported.

### Hypermethylation in *crb2a* ^−/−^ retina

**Figure 4:**
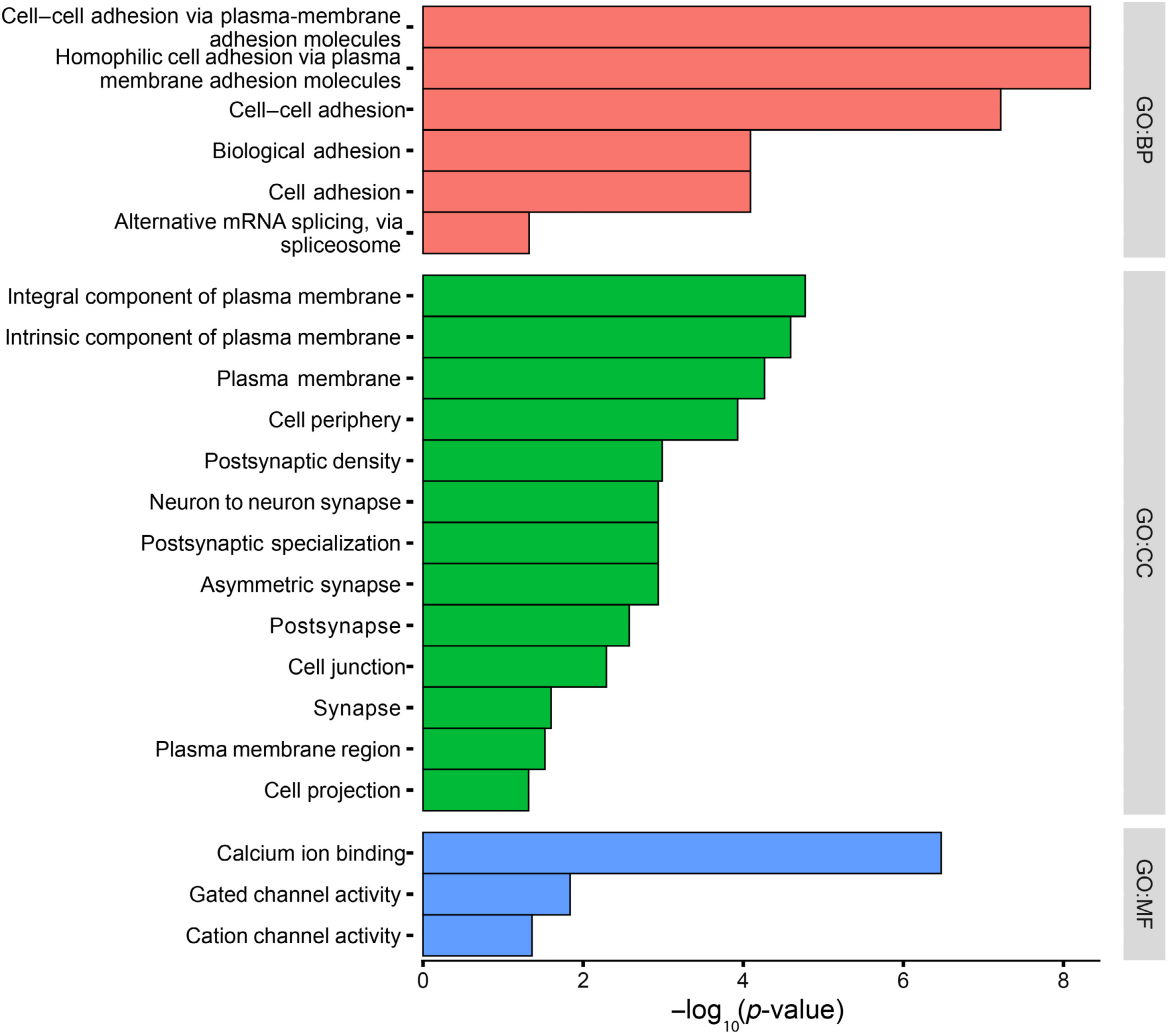
Ontology enrichment analysis of differentially methylated genes (DMGs). Enriched GO analysis of DMGs identified for biological process (BP), cellular compartment (CC), and molecular function (MF).

With increased levels of cellular proliferation and altered expression of genes involved in chromatin modification, epigenetic regulation, and nucleosome assembly, we identified a top DEG, *vezf1a* (LFC 2.14 adjusted *p* value 1.6 × 10^−28^), a DNA‐binding transcription factor whose consensus binding site correlates with CpG islands, so its overexpression in *crb2a* ^−/−^ may indicate transcriptional control in retinal tissue through methylation [53]. We assessed global DNAm, one of the common mechanisms of epigenetic regulation in eukaryotes. We identified 511 genes enriched with at least five DMPs (277 hyper and 234 hypomethylated, supplementary material, Table S3). Ontology analysis for GO:BP of hypermethylated genes indicated enrichment of cell adhesion annotations (including GO:0007156, GO:0098609, GO:0007155, and GO:0022610) involving *ctnna2*, *ncam2*, *dscam*, *itga9*, *nrxn3b nlgn3a*, and *pcdh10a*/*15a* genes (Figure 4). Similarly, enrichment of neuron projection development/morphogenesis and nervous system development annotations were enriched (including GO:0048812, GO:0031175, and GO:0007399) involving *notch1a*, *nos1*, *trarg1a*, *nbeaa*, and *sema5a*. GO:MF analysis identified enrichment of calcium ion binding (GO:0005509), sodium channel activity (GO:0005272), and mitogen‐activated protein (MAP) kinase activity (GO:0004707) with mitogen‐activated protein kinase 6 (*mapk6*) and nemo‐like kinase, type 2 (*nlk2*). GO:CC analysis showed enrichment for cell junction (GO:0030054), cell periphery (GO:0071944), cell projection (GO:0042995), neuron‐to‐neuron synapse (GO:0098984), and plasma membrane (GO:0005886) ontologies.

### Integration with functional epigenetic module (FEM)

**Figure 5:**
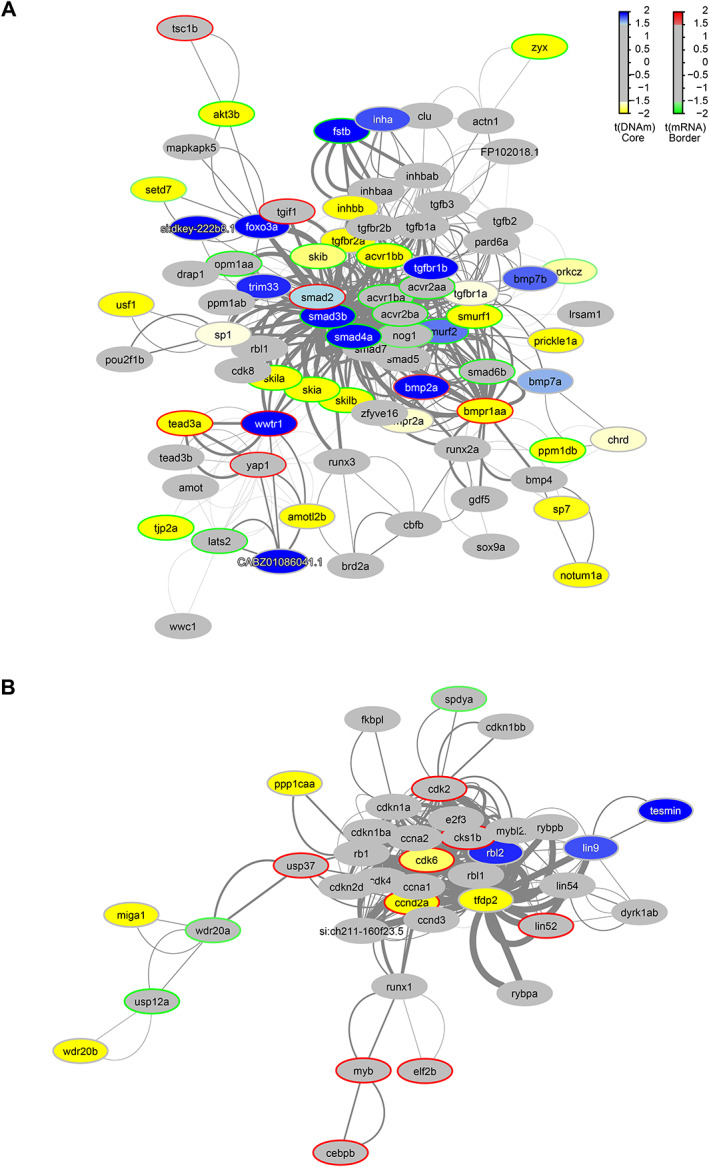
Functional PPI networks. The differentially methylated modules consist of a network of genes based on their functional connectivity using PPI. Each module has a primary gene that is connected to other target genes in the network. Each module was significant *p* < 0.05 using the Functional Epigenetic Module method. GraphNEL networks were exported from the modified FEM R package to Cytoscape (version 3.8) using RCy3 and further annotated. Node colour represents methylation state; blue nodes show hypermethylation, with yellow representing hypomethylation. A different colour of the node border refers to differential expression; those with red borders are upregulated, those with green are downregulated. Edge widths are proportional to the average statistic of the PPI network. (A) The *bmpr1aa/smad3b/smad4a* module is associated with BMP and TGFβ signalling pathways and was reported to be significant through three different seed genes. (B) The *tfpd2/cdk6* module is associated with cell cycle regulation, identified twice with individual seed genes.

To integrate RRBS and RNA‐seq data, we modified the original FEM algorithm code to incorporate data from any species [54]. This is the first report that integrates DNA methylation, RNA‐seq mRNA expression, and the PPI network, highlighting differential expression and methylation controlled either by a single seed gene or a gene hub. Using the zebrafish PPI (StringDB, obtained Nov 2020) as a scaffold, we integrated the RRBS methylation data, gene, and promoter level only, identifying 14 gene hubs and 15 for promoter‐only analysis. Of the expanded gene‐level context, several FEM hubs were identified, with a core set of 12 genes that included *bmpr1aa*, *cdk6*, *smad3b*, *smad4b*, and *tjp2a*. Several networks were identified more than once, including a network of 80 genes identified by three FEM seed genes: *bmpr1aa*, *smad3b*, and *smad4a* (supplementary material, Table S4). Pathway analysis of these hubs (Figure 5A) showed an enrichment of the transforming growth factor β (TGFβ) signalling pathway (WP366), Hippo signalling pathway (Kyoto Encyclopedia of Genes and Genomes), bone morphogenetic protein (BMP) signalling and regulation (WP1425), Hh signalling (WP4249), integrin‐mediated cell adhesion (WP185), TGFBR/BMPR to SMAD signalling, and tight junction assembly. Pathway and ontology analysis of the shared FEM hubs with seed genes *cdk6* and tfdp2 (Figure 5B) highlighted G1 to S cell cycle control (WP45), cell cycle (WP179), and G1/S phase transition, SCF/SKP2 complex, TP53 signalling, and DREAM complex cell cycle progression in cancer. GO:BP analysis identified the negative regulation of cyclin‐dependent protein serine/threonine kinase activity involved in G1/S transition of the mitotic cell cycle, histone phosphorylation, G1/S transition of the mitotic cell cycle, cell cycle G1/S phase transition, and positive regulation of fibroblast proliferation. With two central FEM hubs identified, (i) control of the cell cycle including mitotic G1 phase and G1/S phase transition and (ii) TGFβ signalling, BMP signalling, Hippo signalling, and SMAD protein signal transduction, this has highlighted the epigenetic control of such pathways in *crb2a* ^−/−^ retinal development. We validated the expression of genes from the FEM hubs, using real‐time quantitative RT‐PCR (qRT‐PCR) analysis in ROs. Human orthologues of the genes *bmpr1aa* (*BMPR1A*), *tead3a* (*TEAD3*), *CABZ01086041.1* (*AMOTL1*), *fstb* (*FST*), *yap1* (*YAP1*), and *tjp2a* (*TJP2*) were assessed. The direction of fold‐change correlated between zebrafish and human samples for all targets except *TJP2* and *BMPR1A* (supplementary material, Figure S5A). Validation of qPCR targets in *crb2a* ^−/−^ included the *smad* genes identified within the FEM hubs, as well as *stk3* due to its key role in modulation of the Hippo/YAP pathway (supplementary material, Figure S5B). The increase in *yap1* expression was also confirmed at the protein level (supplementary material, Figure S6), further validating the changes seen in the transcriptome data.

## Discussion

In this study, we reveal novel insights into the molecular pathophysiology of *CRB1*‐related retinal disease through the integration of in‐house multi‐omics data. Gene regulatory network (GRN) analysis confirmed known roles of CRB1 but also revealed novel misregulation of epigenetic control as a pathological mechanism.

One canonical role of the crumbs complex is the formation of epithelial zonula adherens (ZA), which when disrupted leads to OLM cellular adhesion defects ([36, 37] for reviews). We have shown that loss of *crb2a* has an impact on GRNs governing cell cycle control and progression, with a strong effect on the methylation signature of the developing retina. The increased expression of transcription factors pax6 and vsx2 heralds the maintenance of an expanded population of early RPCs, and the lack of photoreceptors is not due to increased apoptosis but a prolonged proliferative cell state, resulting in the thickened and delaminated retinal structure seen in patients. We propose that the retinal changes seen within *crb2a* ^−/−^ result from a far more complicated interplay of gene expression and methylation states than previously considered. This is the first study to provide detailed analysis of the transcriptome for *CRB1* retinopathy with corresponding correlation to the epigenome.

The multi‐omic analysis of *crb2a* ^−/−^ retina highlighted enriched pathways involving cellular adhesion, ZA, and cell‐to‐cell communication, as expected [36]. We demonstrated that genes involved in membrane disruption were overexpressed in the disease state. However, we also found molecular signatures involved in cell cycle control and progression were significantly enriched and had not been thoroughly explored in this disorder previously. Core to the mammalian Hippo pathway are kinases MST1/2 and LATS1/2 and the transcriptional activator YAP that acts through coactivators such as the TEAD family of transcription factors. We identified downregulation of *stk3* (ortholog of *MST2*) in *crb2a* ^−/−^ retina, with increased expression of *yap1*, promoting progenitor cell proliferation, survival, organ growth, and chromatin remodelling, supporting the role of crumbs regulation of the Hippo/YAP pathway [55]. Retinal specific overexpression of yap1 results in abnormal retinogenesis and reduced cell‐cycle exit [56, 57]. Transcriptional regulation pathways were significantly upregulated with increased expression of *tead1b*, thereby adding further evidence of a maintained proliferative state. Misregulation of YAP by the reduction of PALS1 or Crumbs has been shown to result in loss of cell differentiation and tissue morphogenesis defects in renal and airway epithelial cells [58]. Heterozygous‐dominant *YAP1* variants cause developmental eye disorders, including microphthalmia and coloboma [59, 60, 61]. The paired‐box gene family play significant roles in retinal neurogenesis with both *Pax6* and *Pax2* initially expressed in the optic vesicle, followed by downregulation of *Pax2* in the neural retina with localisation to the optic stalk [62]. We found a significantly increased level of *pax2*, with a concomitant increase of *pax6*, indicative of a lack of cellular differentiation. Conditional deletion of Pax6 in RPC has shown increased cell‐cycle exit and coregulation of Vsx2 [51]. Upon querying a single cell RNA‐seq dataset, we showed *pax2* highly expressing cell types clustered with early progenitor cells [63].Therefore, there remains the possibility that interplay between *PAX2/6* and *YAP1* influences the proliferative state seen in *CRB1* retinopathy. *YAP1–MAMLD1* gene fusion‐driven neuroepithelial tumours demonstrating hyperproliferation showed an increase in Yap1 expression, which correlated with an increase in Pax6 [64].

We identified several GRNs, including chromatin remodelling/organisation and epigenetic regulation, leading us to investigate the retinal epigenetic profile of *crb2a* ^−/−^. There is an inheritable epigenetic signature facilitated through DNA methylation, although our understanding in the retina remains limited [65]. The multi‐omic integrative analysis of *crb2a* ^−/−^ identified 280 genes that possessed a concordant gene expression and DNA methylation pattern, highlighting a novel underlying pathological mechanism. Key concordant genes included *smad3b*, *fstb*, *tead3a*, *smad4a*, *bmpr1aa*, and *amotl1*, which are key transcriptional regulators in the TGFβ, BMP, and nodal signalling pathways. TGFβ is a major inductor of epithelial–mesenchymal transition (EMT) during cell fate determination, proliferation, fibrosis, and carcinogenesis and is characterised by the loss of cell–cell contacts, lack of polarisation, remodelling of the actin cytoskeleton, and separation of cells. Although these are in line with the loss of *crb2a* in the retina, no reports have shown EMT within the neural retina, and only recently did a *CRB2*‐related RP variant demonstrate EMT of ARPE‐19 cells [21]. As shown in murine breast epithelial cells, the Hippo pathway can drive the cytoplasmic localisation of TAZ/YAP, sequestering SMAD complexes, resulting in the suppression of TGFβ signalling [66, 67]. The exact interplay of these molecules within the retina remains to be further elucidated, specifically the involvement of epigenetic regulation. Activation of the TGFβ pathway correlates with increased proliferation of zebrafish Müller glia. Chemical inhibition of TGFβ type I receptors using SB431542 resulted in increased cell proliferation in the ONL after induced retinal degeneration [68]. Modulation of this pathway may act as a possible therapeutic target for *CRB1*‐retinopathy. Further uncoupling of expression and chromatin state will provide direct insight into the molecular changes in the retina, possibly via Assay for Transposase‐Accessible Chromatin using sequencing (ATAC‐seq) [69] or Hi‐C‐seq [70].

Retinal imaging using spectral domain optical coherence tomography (SD‐OCT) has revealed distinct differences in the retinal structure of *CRB1* patients. Those with a LCA phenotype have no identifiable photoreceptors in the ellipsoid zone (EZ), an undetectable external limiting membrane (ELM), and a severely attenuated or unidentifiable ONL [11]. In contrast, patients with RP have a granular or attenuated EZ affecting the macula with foveal sparing and a similar ELM but normal to attenuated ONL. Our patient had a compound missense variant in *CRB1* c.2548G>A p.(Gly850Ser) and splice site variant (c.4006‐10A>G) and critically showed no CRB1 or MPP5 protein expression. This contrasted other ROs derived from *CRB1*‐related RP patients with (i) homozygous missense c.3122T>C, p.(Met1041Thr) variants and (ii) compound heterozygous nonsense c.2983G>T, p.(Glu995*) and missense c.1892A>G, p.(Tyr631Cys) variants, both of which showed correct localisation of Crumbs cell polarity complex proteins, including MPP5. Mutant CRB1 localised to the subapical region above the ZA, as in controls, but also to the neuroblast layer and ONL of the ROs [12]. These differences likely contribute to clinical heterogeneity and must be considered when translating new gene therapies to patients. Those with LCA and no identifiable photoreceptors may require a different therapeutic strategy due to the maldeveloped retinal architecture. A better understanding of the human molecular disease mechanisms will lead to insights regarding potential novel therapeutic targets. Further work is required to understand the genotype–phenotype correlation, comparison of RNA‐seq datasets from *CRB1*‐RP ROs versus those derived from LCA patients may provide deeper insights into the disease's pathophysiology.

In summary, we have highlighted the interplay of DNA methylation and gene regulation in the *crb2a* ^−/−^ retina, advancing our understanding of the mechanisms underlying *CRB1*‐associated eye disease. Further targeted analysis of retinal cell migration, proliferation, differentiation, and cell cycle control through single‐cell multi‐omic approaches, both spatially and longitudinally, will provide deeper insights into disease pathophysiology. In addition, investigation of other pathogenic variants causing the various *CRB1* retinal phenotypes will shed light on differences in disease patterns and aid the identification of potential therapeutic targets that are lacking for these conditions.

## Author contributions statement

MM conceptualised and supervised the project and provided resources. MT, LT, RR, DTW and RY conducted experimental procedures. NO and YT undertook the bioinformatics analysis and visualisation. NO wrote the original draft manuscript. MM, MT, LT, RR, RY, YT and SB revised it after review. All authors read and approved the final manuscript and had access to and verified all underlying data.

## Supporting information


**Figure S1.** Characterisation of retinal histology of *crb2a* ^*−/−*^ model
**Figure S2.** Global analysis of RNA‐seq data of retina from zebrafish *crb2a* ^*−/−*^
**Figure S3.** Expression of Crumbs protein complex‐associated genes in *crb2a* ^*−/−*^ retina in zebrafish
**Figure S4.** Expression of mpp5 in zebrafish retina at 56 hpf
**Figure S5.** Validation of FEM hub genes
**Figure S6.** *crb2a* ^*−/−*^ shows increased expression of yap1 in retina
**Table S1.** qPCR targets and primer sequences

**Table S2.** RNA‐seq differentially expressed genes

**Table S3.** Differentially methylated CpGs in zebrafish *crb2a* ^*−/−*^ retina

**Table S4.** Summarisation of identified functional epigenetic modules calculated between *crb2a* ^*−/−*^ and WT retina

## Data Availability

The datasets presented in this study were deposited in the Gene Expression Omnibus (NCBI GEO) database, under accession numbers GSE178842 (RRBS) and GSE178709 (RNA‐seq). Data analysis scripts can be found at https://bit.ly/crb-omics. All other relevant data supporting the key findings of this study are available within the article and its Supplementary Information files or from the corresponding author upon reasonable request.
