# Supplementary material for: Loss of the crumbs cell polarity complex disrupts epigenetic transcriptional control and cell cycle progression in the developing retina
Source: J Pathol. 2023 Feb 9;259(4):441–54. doi: 10.1002/path.6056 (PMC10601974; doi:10.1002/path.6056)
Supplement: Supplementary file 1 — Figure S1. Characterisation of retinal histology of crb2a −/− model Figure S2. Global analysis of RNA‐seq data of retina from zebrafish crb2a −/− Figure S3. Expression of Crumbs protein complex‐associated genes in crb2a −/− retina in zebrafish Figure S4. Expression of mpp5 in zebrafish retina at 56 hpf Figure S5. Validation of FEM hub genes Figure S6. crb2a −/− shows increased expression of yap1 in retina Table S1. qPCR targets and primer sequences [file PATH-259-441-s002.docx]

**Loss of the crumbs cell polarity complex disrupts epigenetic transcriptional control and cell cycle progression in the developing retina**

N Owen *et al. J Pathol* <https://doi.org/10.1002/path.6056>

**Supplementary Figures S1–S6**

**Supplementary Tables S1**

**Supplementary Tables S2–S4 are provided as separate Excel files**


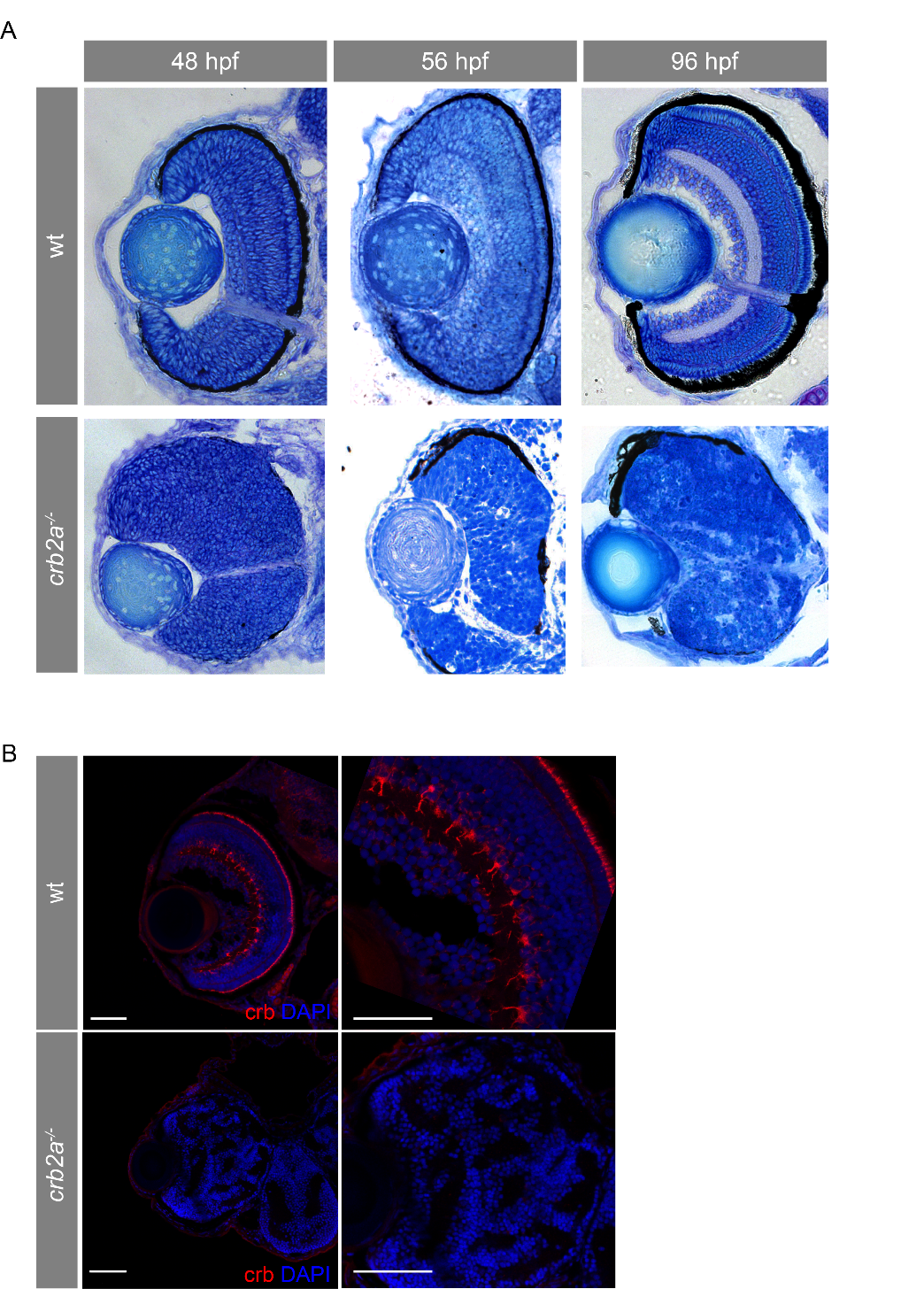


**Figure S1. Characterisation of retinal histology of *crb2a*^−/−^ model.** (A) Retinal plastic sections of zebrafish *crb2a*^−^*^/−^* eye at 48, 56, and 96 hpf compared to WT eyes at the same time points, stained with toluidine blue. (B) IHC analysis of expression of crb protein in WT and *crb2a*^−^*^/−^* zebrafish, with magnified representative region. Scale bar, 50 μm.
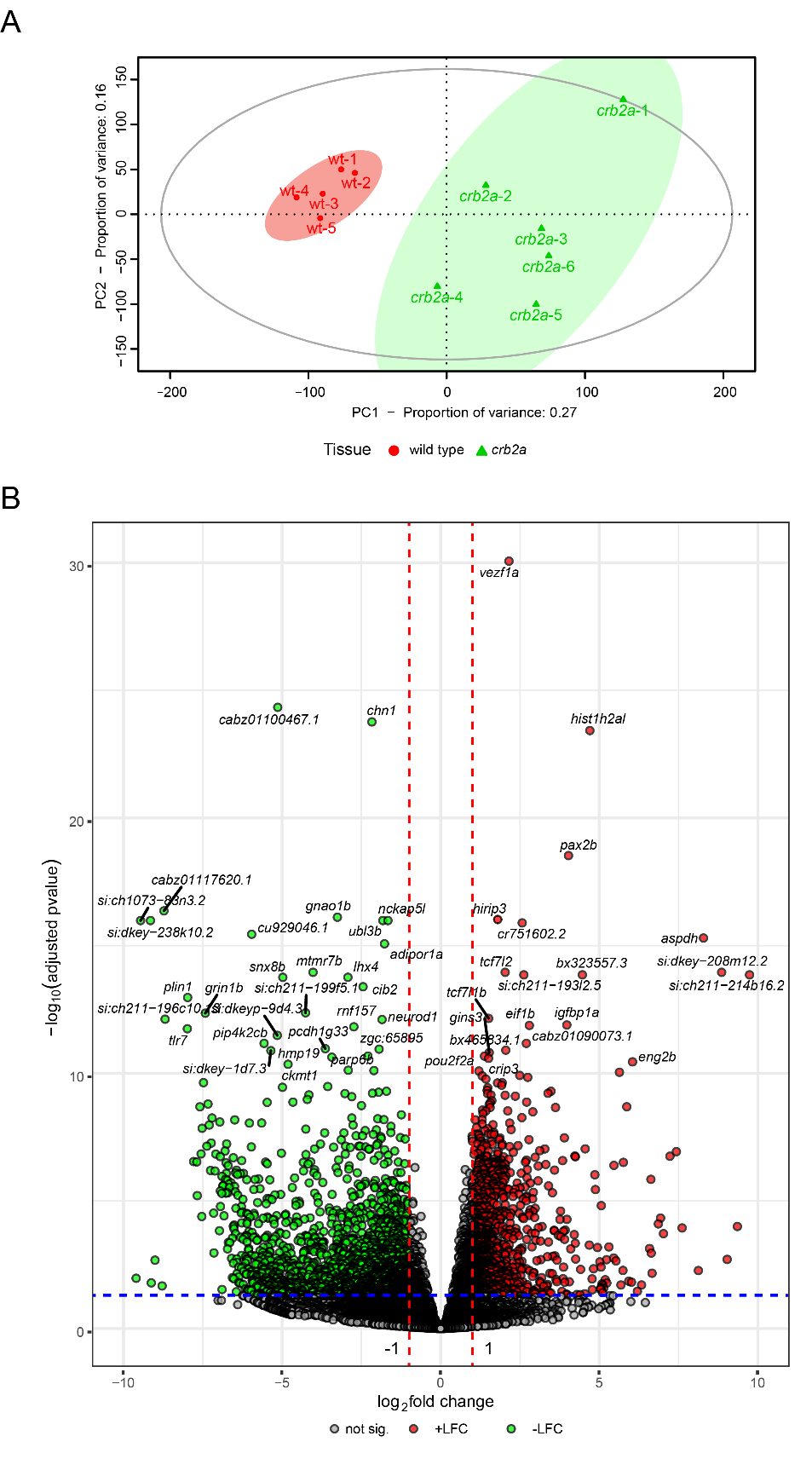


**Figure S2.** **Global analysis of RNA-seq data of retina from zebrafish *crb2a^−/−^*.** (A) Principal component (PC) analysis of mean-centred and scaled rlog-transformed read count values indicating largest source of variation among samples was tissue origin, accounting for 60% of variation in data. (B) Volcano plot of differentially/nondifferentially expressed genes between *crb2a*^−/−^ and WT retina. DEGs were identified using thresholds for adjusted *p* value (≤ 0.05), log2 fold-change ≥ 1 for upregulated genes (red), ≤ −1 for downregulated genes (green). The top 50 significant genes are highlighted.


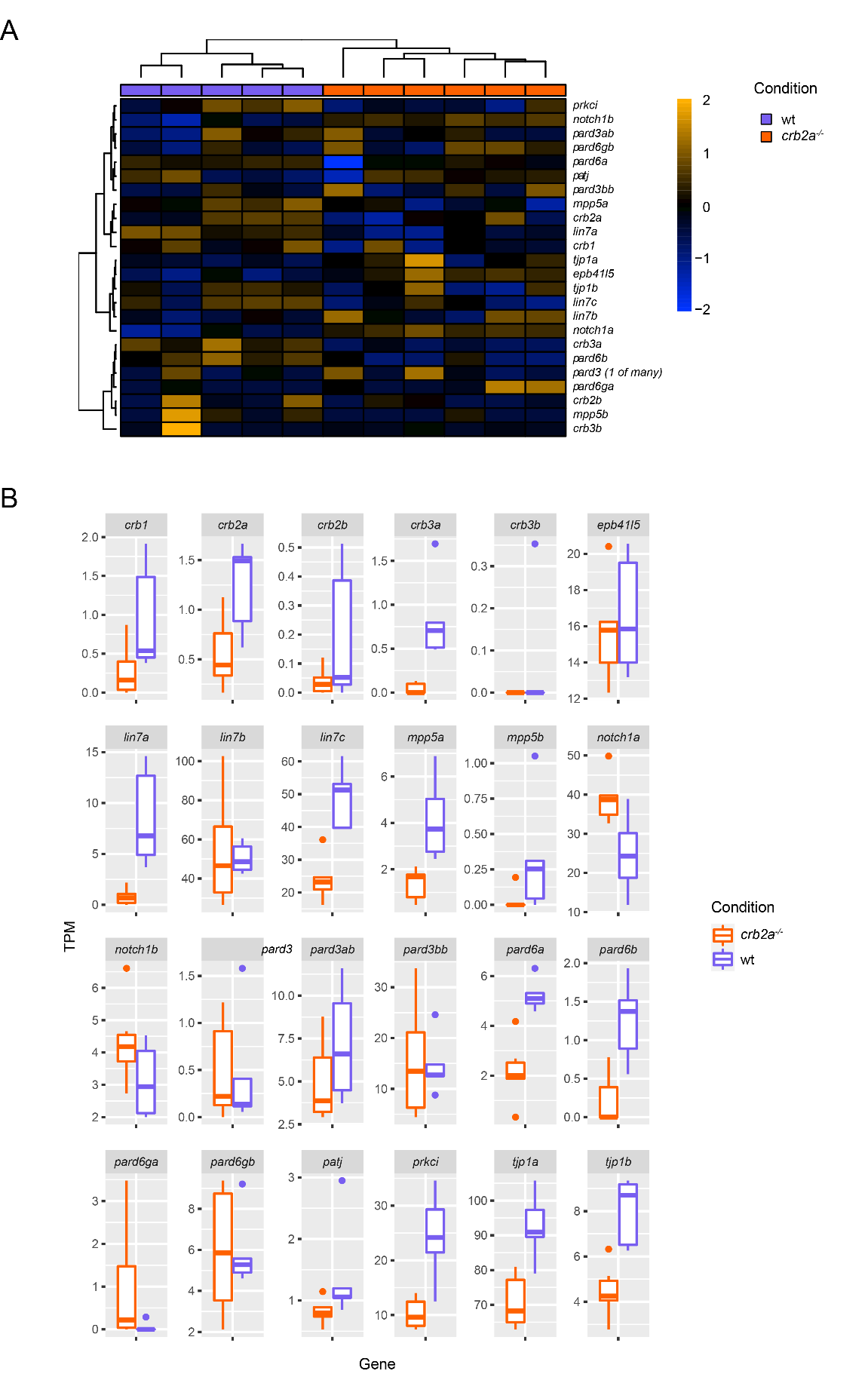


**Figure S3. Expression of crumbs protein complex associated genes in *crb2a^−/−^* retina in zebrafish.** (A) Heatmap of *z*-scores of expression of representative crumbs cell polarity complex-related genes in *crb2a****^−^****^/^****^−^*** retina showing upregulation of *notch1* and absence of *mpp5*. (B) Expression of *CRB1*-associated genes in zebrafish expressed as transcripts per kilobase million (TPM).


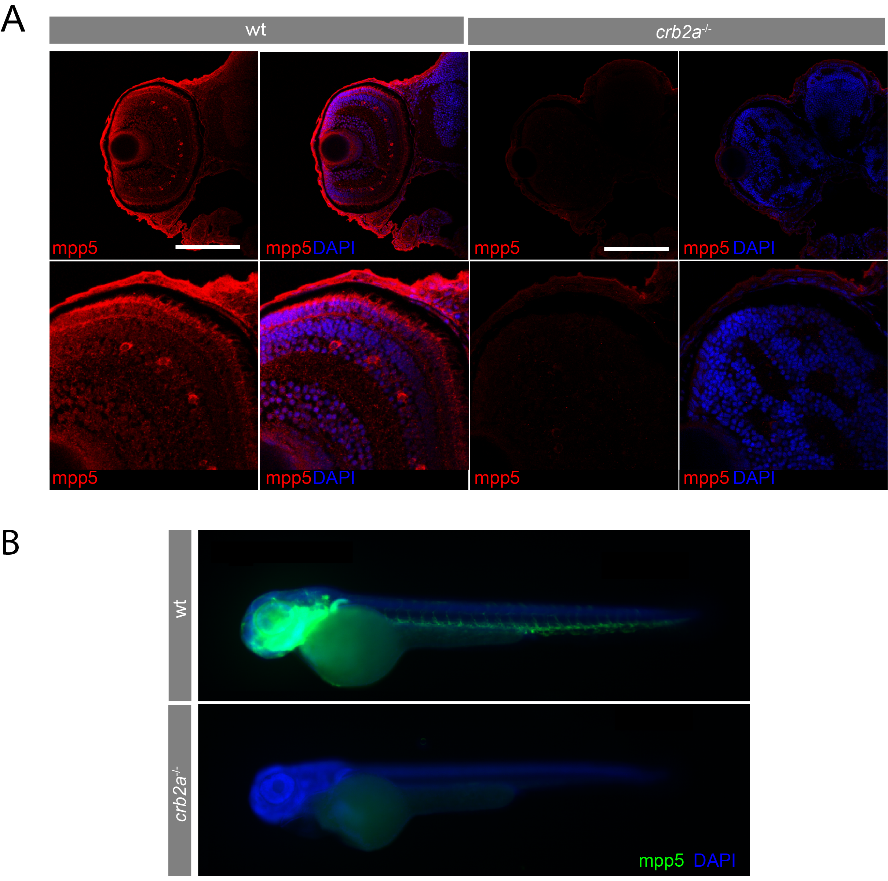


**Figure S4.** **Expression of mpp5 in zebrafish retina at 56 hpf.** (A) Retinal mpp5 expression detected using anti-mpp5 antibody showing distinct localisation in horizontal cells (WT) compared to absence of expression in *crb2a^−/−^* retina. Magnified regions show representative 100-µm zoom. (B) Whole mount detection of mpp5 in zebrafish embryos at 96 hpf showing an absence in *crb2a^−/−^* model compared to WT. Scale bar, 100 µm.


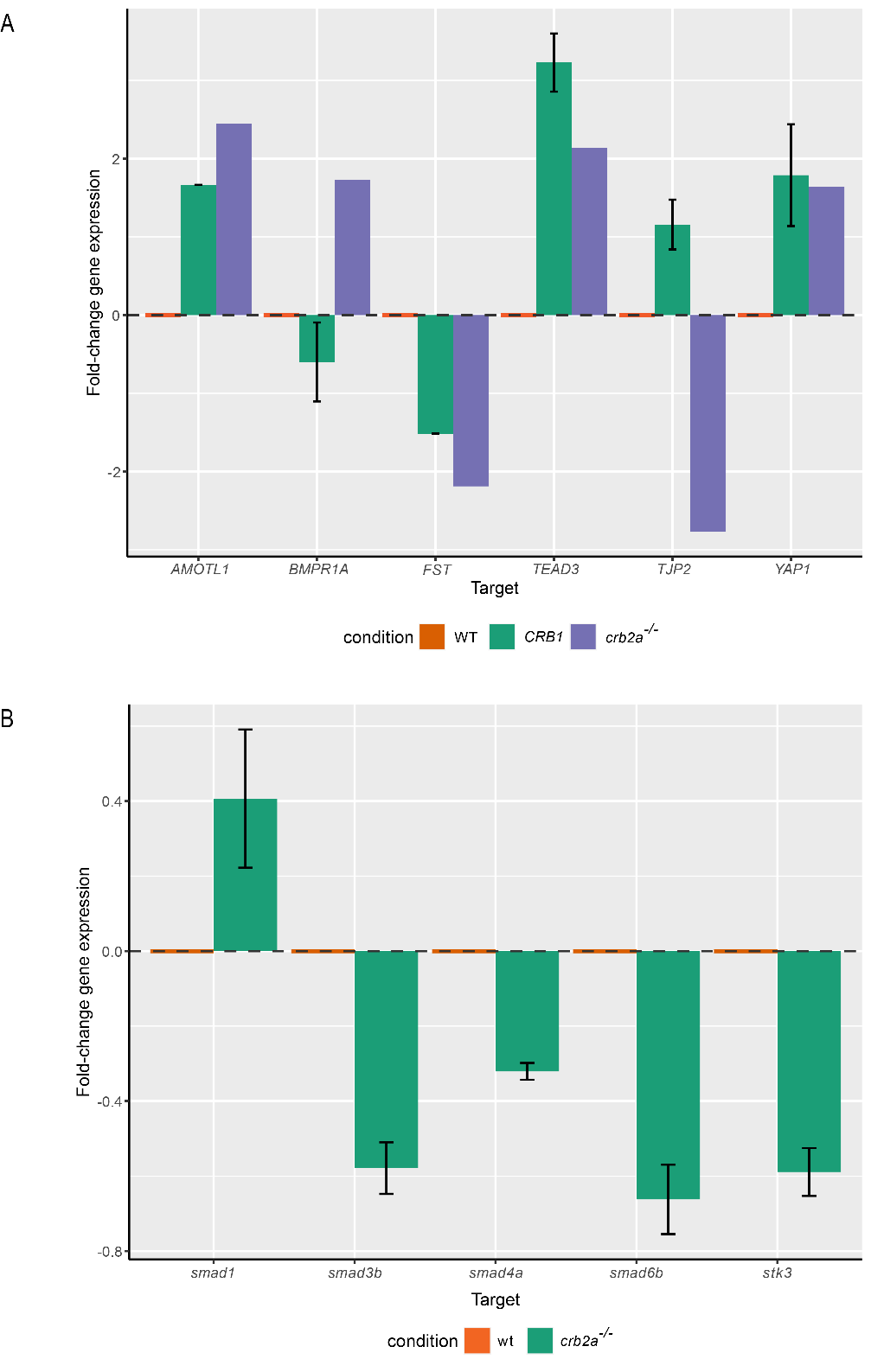


**Figure S5. Validation of FEM hub genes.** (A) qPCR validation of expression of selected genes from FEM hubs in ROs. Human orthologues of genes *bmpr1aa* (*BMPR1A*), *tead3a* (*TEAD3*), *CABZ01086041.1* (*AMOTL1*), *fstb* (*FST*), *yap1* (*YAP1*), and *tjp2a* (*TJP2*) were assessed (*n* = 3). Direction of fold-change correlated between zebrafish and human samples for all targets except *TJP2* and *BMPR1A*. (B) qPCR validation of expression of selected hub genes in zebrafish retina at 56 hpf, *crb2a^−/−^* compared to WT controls (*n* = 4).


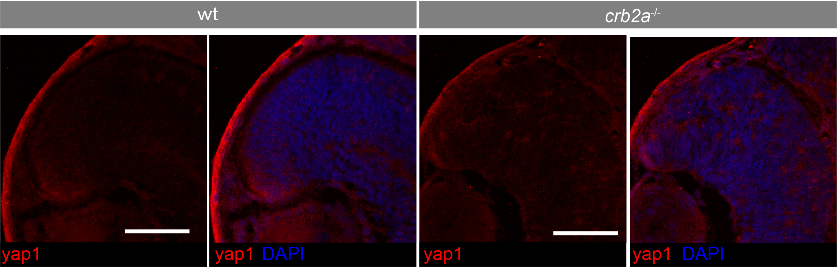


**Figure S6.** ***crb2a^−/−^* shows increased expression of yap1 in retina.** Retinal yap1 expression detected using anti-yap1 antibody showing increased expression in *crb2a^−/−^* retina as compared to WT retina, at 56 hpf. Scale bar, 50 µm.

**Table S1. Primers used for qPCR validation**

| **Target** | **Gene symbol** | **Accession** | **Forward Primer** | **Reverse Primer** |
| --- | --- | --- | --- | --- |
| Glyceraldehyde-3-phosphate dehydrogenase | *GAPDH* | NM_002046.7 | GCTGCATTCGCCCTCTTA | GAGGCTCCTCCAGAATATGTGA |
| Bone morphogenetic protein  receptor type 1A | *BMPR1A* | NM_004329.3 | CATACTTGGTTTCATAGCGG | ATAAGCCAATTTAAGCAGGG |
| TEA domain transcription factor 3 | *TEAD3* | NM_003214.4 | CCCCACCTAGGTTTTATAATG | TTTTTACATGACTGACCCAC |
| Tight junction protein 2 | *TJP2* | NM_004817.4 | CAAGATCAATGGGACTGTAAC | GCTGTAGTTTTCCTCTTGAC |
| Yes1 associated  transcriptional regulator | *YAP1* | NM_001130145.3 | AATTGAGAACAATGACGACC | AGTATCACCTGTATCCATCTC |
| Angiomotin like 1 | *AMOTL1* | NM_130847.3 | TAGAAGACTCCACCTACTTTTC | AACAACTTTTTCCCTGATGC |
| Follistatin | *FST* | NM_013409.3 | TAAAGAAACGTGTGAGAACG | CTTTACATCTTGCCTTTAGGAG |
| SMAD family member 3b | *smad3b* | NM_175083.2 | GACACAGGTTCTCCAACGCT | GAAAGTCTCACCGACACGCT |
| SMAD family member 4a | *smad4a* | NM_001122700.1 | GGCCAGACCTCCACATACCA | AGTATTCAGGAGCAGGGTGGTT |
| SMAD family member 6b | *smad6b* | NM_001045051.2 | AGACTTTCCCCCAGCGAAGA | TCCCAGTACGCCACGTTACA |
| SMAD family member 1 | *smad1* | NM_131356.2 | GCCCTAGAGTGCTGTGAGTT | GCAGCATGGAGAGTTTAGCG |
| Serine/threonine kinase 3 | *skt3* | NM_199672.1 | GGGACATCAAAGCCGGGAAT | CTGCCACACAGTTGTACCCA |
| Ribosomal protein L13a | *rpl13a* | NM_212784.1 | TCTGGAGGACTGTAAGAGGTATGC | AGACGCACAATCTTGAGAGCAG |

**Tables S2–S4 are provided as separate Excel files.**
